# Spinal granulocytic sarcoma with both extradural and intradural involvement: a case report

**DOI:** 10.3389/fonc.2026.1832811

**Published:** 2026-09-16

**Authors:** Jian Zhang, Bao-Guo Han, Yan-Hao Ge, Ting-Chen Zhu, Ya-Feng Zhang

**Affiliations:** 1 Nanjing University of Chinese Medicine, Nanjing, Jiangsu, China; 2 Wuxi Traditional Chinese Medicine (TCM) Hospital Affiliated to Nanjing University of Chinese Medicine, Wuxi, Jiangsu, China; 3 Wuxi Xishan District Hospital of Traditional Chinese Medicine, Wuxi, Jiangsu, China

**Keywords:** granulocytic sarcoma, intradural, laminectomy, leukemia relapse, nerve sheath tumors

## Abstract

**Introduction:**

Granulocytic sarcoma (GS) is a rare extramedullary accumulation of myeloid blasts. Cases of GS with isolated intradural presentation sparing the underlying bone are extremely rare.

**Case description:**

A 75-year-old male patient with a remote history of acute myeloid leukemia (AML) in 30-year remission presented with progressive lumbar radiculopathy. Magnetic Resonance Imaging (MRI) revealed intradural lesions spanning L2–S1, mimicking nerve sheath tumors. The patient underwent emergency laminectomy and resection of a vascular, grayish-green L2 mass adherent to the cauda equina. Immunohistochemistry confirmed it was a GS (MPO+, CD43+, CD68+) with no evidence of bone marrow relapse. Following surgery and consolidation chemotherapy, the patient’s neurological status improved (ASIA C to D). However, he developed disseminated CNS relapse four months later and succumbed to disease progression 7 months postoperatively.

**Conclusion:**

Isolated intradural GS constitutes a rare manifestation of extramedullary AML relapse and frequently masquerades radiologically as common benign nerve sheath tumors. While urgent surgical decompression facilitates significant neurological recovery, long-term survival is strictly dictated by the efficacy of systemic disease control. Therefore, adopting a multidisciplinary strategy integrating intraoperative frozen-section analysis, rigorous immunohistochemical profiling, and early adjuvant chemotherapy is paramount for avoiding diagnostic delays and optimizing patient outcomes.

## 1 Introduction

Granulocytic sarcoma (GS), also designated as myeloid sarcoma, is an uncommon extramedullary tumor composed of immature granulocytes or myeloid blasts. Although frequently concomitant with systemic hematologic malignancies or myeloproliferative neoplasms, isolated spinal cord involvement remains an exceedingly rare clinical entity. Such rarity presents substantial challenges regarding timely diagnosis and the implementation of optimal therapeutic strategies (1).

## 2 Case description

A 75-year-old male patient presented with a 1-month history of progressive lower back pain, accompanied by radiculopathy, paresthesia, and functional impairment of the left lower extremity. The patient reported that the pain radiated from the left gluteal region to the anterior thigh and calf, with associated numbness and subjective weakness. His medical history was notable for acute myeloblastic leukemia (AML) 30 years ago, which achieved remission after chemotherapy. He had no smoking, drinking, or radiation exposure history, and no family history of malignancy. Physical examination revealed tenderness in the left gluteal and inguinal regions. The patient’s vital signs were stable, and no significant abnormalities were detected on cardiopulmonary or abdominal examination. Orthopedic and neurological assessment revealed an antalgic gait; spinal inspection showed no obvious scoliosis or kyphosis with preserved lumbar lordosis. Tenderness was elicited at the L4-L5 and L5-S1 interspinous and paravertebral regions, more pronounced on the left, and localized deep tenderness over the left gluteal and groin areas provoked radiating pain to the anterior thigh and lower leg. On neurological examination, sensory testing demonstrated markedly diminished pinprick and light touch in the left L2, L3, and L4 dermatomes compared to the contralateral side, while L5 and S1 sensation was relatively preserved. Motor examination revealed left-sided weakness: iliopsoas 4/5, quadriceps femoris 4+/5, with other muscle groups 5/5 bilaterally. Reflexes showed significantly diminished left patellar and Achilles tendon reflexes; the left Babinski sign was negative and no ankle clonus was observed. The straight leg raise test was negative bilaterally, but the femoral nerve stretch test was positive on the left, reproducing anterior thigh pain. Distal blood perfusion and trophic status of the bilateral lower extremities were unremarkable. Sensation was decreased in the corresponding dermatomes. Laboratory tests revealed elevated hs-CRP (12.38 mg/L) and mildly reduced albumin (34.6 g/L). Renal function was mildly impaired (eGFR 61.7 mL/min/1.73m²) with elevated uric acid (464 μmol/L), urea (10.2 mmol/L), and cystatin C (1.46 mg/L). Peripheral blood morphology showed no blast cells, and tumor markers were within normal limits. Lumbar spine MRI (**Figure 1**) demonstrated multiple well-defined intraspinal extramedullary masses from L2 to S1 levels, along with L1-S1 intervertebral disc deformation and bulging. Based on clinical and imaging findings, the patient was diagnosed with symptomatic intraspinal occupying lesions at multiple levels causing nerve root compression. Histopathological confirmation was recommended to rule out hematologic recurrence.

**Figure 1:**
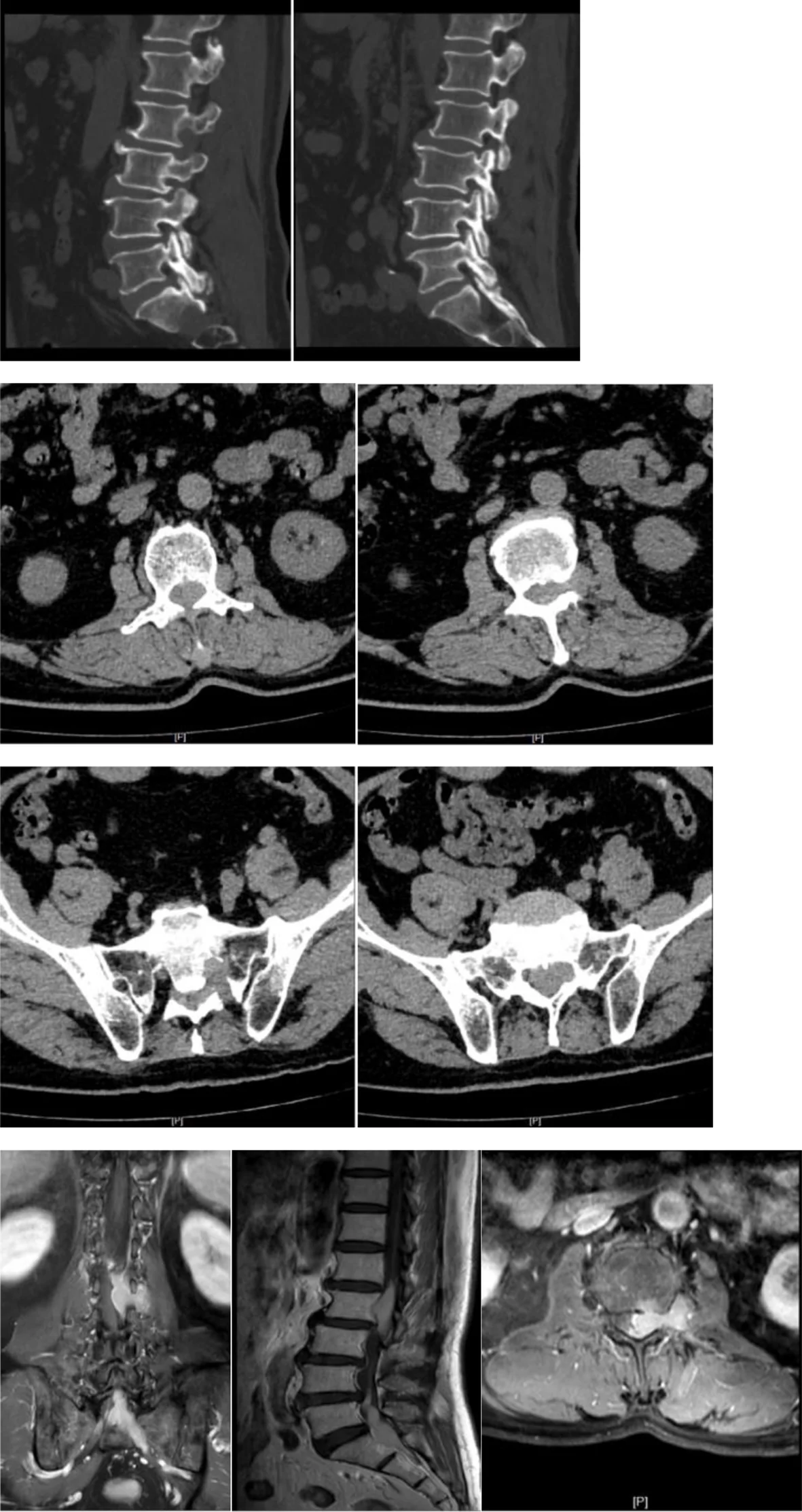
Sagittal T2 magnetic resonance imaging (MRI) show severe spinal cord compression at L2–S1, axial T2 MRI show severe spinal cord compression at L2–S1.

Based on the patient’s clinical signs and imaging findings, the symptoms were attributed to compression caused by an intraspinal space-occupying lesion at the L1-L3 levels. On September 1, 2023, the patient underwent a posterior L1/L2 and L2/L3 laminectomy for decompression, L2 intraspinal tumor resection, and pedicle screw internal fixation under general anesthesia with endotracheal intubation.

After induction of anesthesia, the patient was placed in the prone position. Following routine skin preparation and sterile draping, a posterior midline incision of approximately 12 cm was made. The subcutaneous tissue and deep fascia were incised sequentially. Bilateral paraspinal muscles were stripped along both sides of the spinous processes to fully expose the bilateral laminae and facet joints from L1 to L3. The spinous processes of L1 and L2 were resected. Subsequently, using a high-speed drill and laminectomy rongeurs, the laminae of L1 and L2 were resected along the medial base of the bilateral facet joints, and the superior margin of the L3 lamina was partially removed to expand the surgical field. The resected bone fragments were trimmed into fine granules, mixed with a bioactive glass bone graft substitute, and set aside for subsequent bone grafting.

After the ligamentum flavum was resected, the dural sac and bilateral L1/L2 nerve roots were fully exposed. The dural sac at the L2 level was visibly distended and tense. The thickened nerve root sheath dura was incised to enter the intradural space, and the dural edges were retracted bilaterally and secured with stay sutures.

Exploration revealed an oval-shaped intradural tumor measuring approximately 3 × 1 × 1 cm. The tumor was located slightly to the left within the spinal canal, with rich vascularity and tortuous vessels, and was densely adherent to a portion of the cauda equina. The tumor extended from the L1 nerve root into the extradural space.

After carefully dissecting the cauda equina nerves densely adherent to the tumor and achieving meticulous hemostasis, the solid intradural portion was mobilized en bloc as a single tumor. Re-exploration confirmed adequate relaxation of the cauda equina and a watertight dural closure. The medial portion of the left L2 superior articular process was resected. Further exploration revealed a gelatinous tumor attached to the origin of the left L1 nerve root extradurally, representing an extradural extension of the intradural tumor via the L1 nerve root. The gelatinous portion of the tumor was carefully dissected from the adherent nerve root. The completely resected tumor specimen and the gelatinous material were sent for pathological examination to determine the nature of the components in different regions of the lesion.

The surgical field was thoroughly irrigated with normal saline. Exploration confirmed no active bleeding, and the dural sac exhibited good pulsation. The bilateral intertransverse bone surfaces at L1/L2 and L2/L3 were decorticated using a high-speed drill. The previously prepared mixture of fine bone granules and bioactive glass bone graft substitute was implanted. L1 pedicle screws were placed bilaterally, a right L2 pedicle screw was placed unilaterally, and L3 pedicle screws were placed bilaterally, followed by rod installation and fixation. Due to tumor erosion and intraoperative resection of the facet joint resulting in suboptimal conditions for screw placement, the left L2 screw was omitted. Intraoperative C-arm fluoroscopy confirmed satisfactory positioning of the remaining 5 screws and connecting rods, with adequate overall stability of the internal fixation construct.

The incision was irrigated again with normal saline, and a negative pressure drainage tube was placed on the left side. The incision was closed in layers. The procedure was concluded, the wound was dressed, and the patient was fitted with a lumbar brace for external fixation.

The excised tumor, appearing gray-green (**Figure 2**), was sent for pathological evaluation. Sterile dressings were applied.

**Figure 2:**
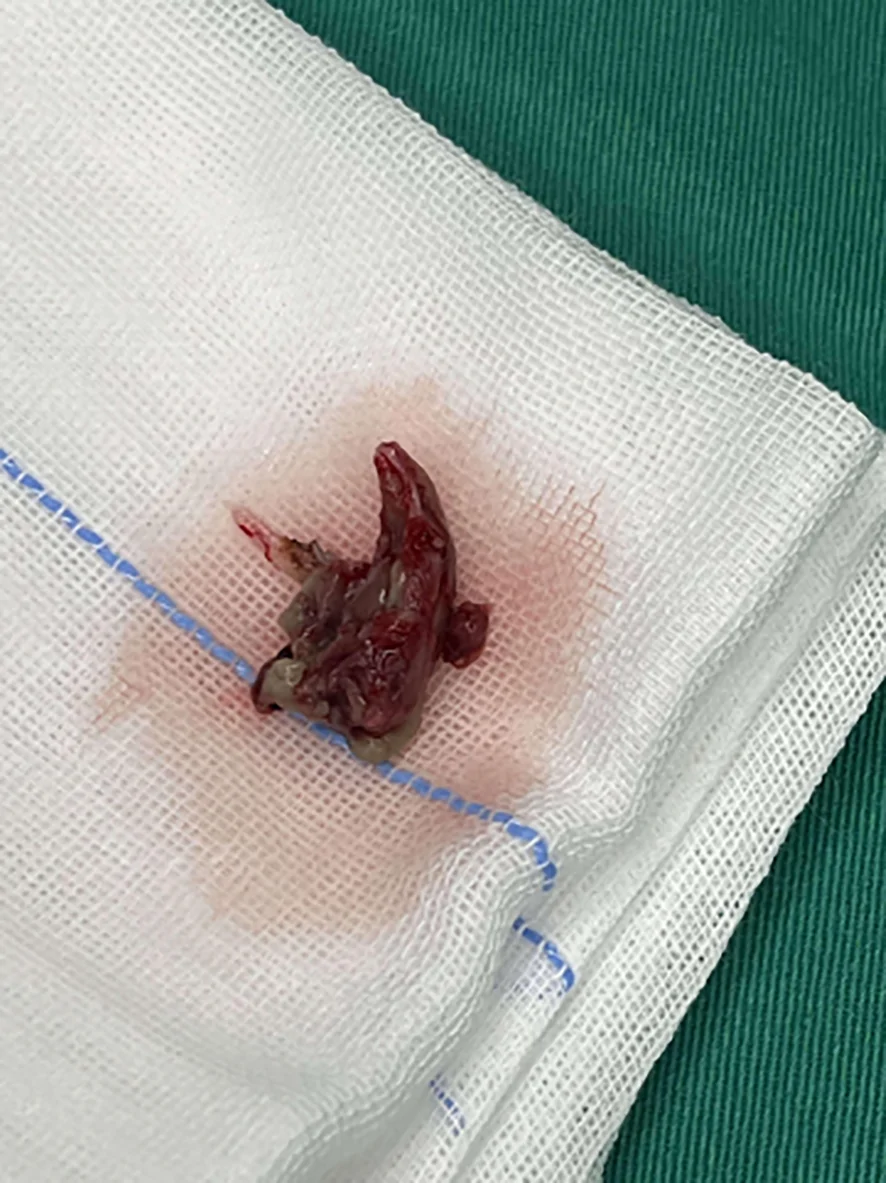
An oval, olive-gray, tumor measuring approximately 3 × 1 × 1 cm was removed from the spinal canal during surgery.

By postoperative day 6, the patient was ambulatory with lower extremity strength improved to MRC grade 4/5. Neurological examination revealed hyperactive patellar and Achilles reflexes, but diminished cremasteric and anal reflexes, with a negative Babinski sign. Spinal imaging demonstrated expected postoperative changes (**Figure 3**).

**Figure 3:**
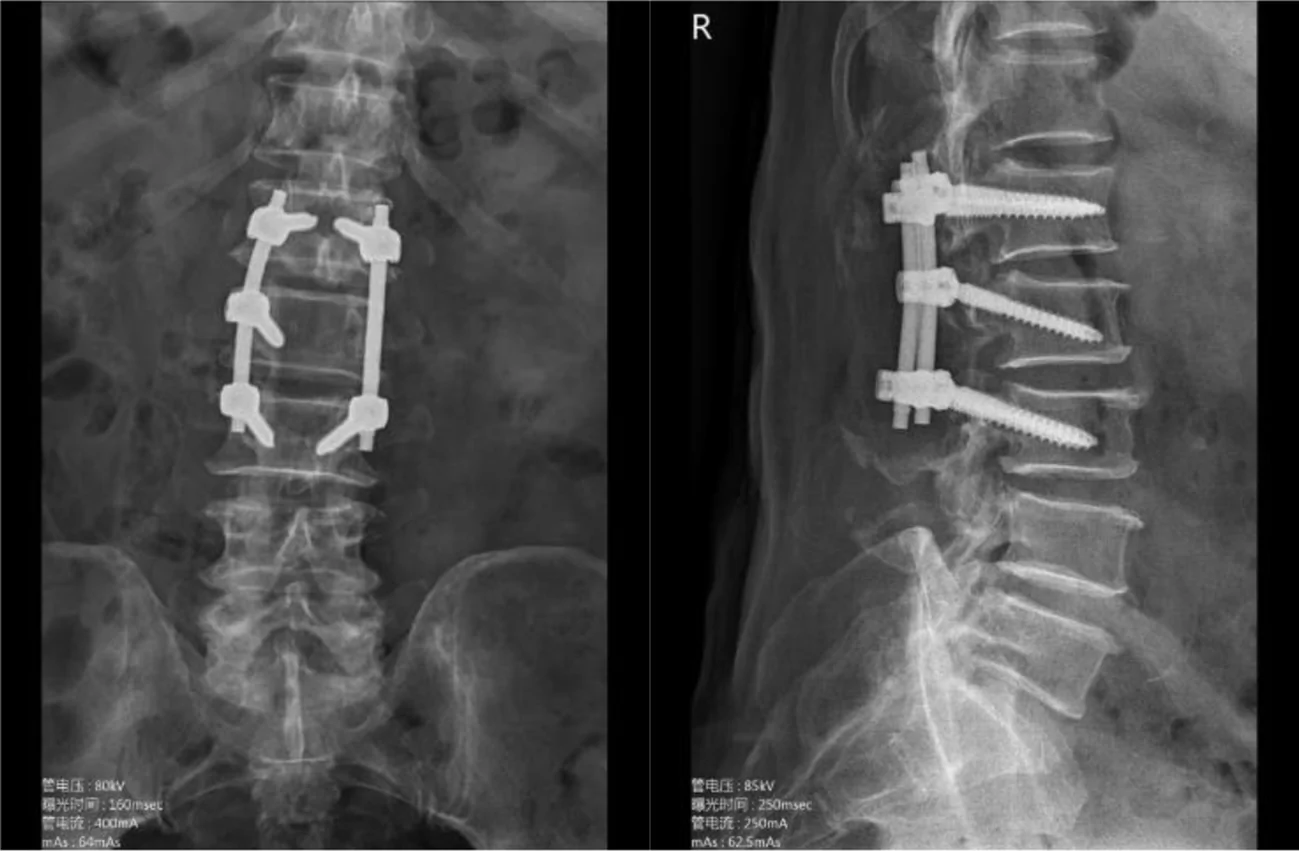
Postoperative X-rays showed postoperative changes and degenerative changes of the lumbar spine.

The final diagnosis, based on the pathological evaluation, was an intraspinal myeloid tumor consistent with sarcoma or leukemic infiltration (**Figure 4**). Histological examination showed tumor cells that were positive for CD68, Bcl-2, CD43, and myeloperoxidase (MPO), with partial positivity for Lysozyme and CD117. Scattered S-100 positivity was also observed. Conversely, the cells were negative for lymphoid markers (CD3, CD20, CD79a, PAX-5), plasma cell markers (CD138, CD38), epithelial markers (CK, EMA), and melanocytic markers (HMB45, SOX10). The Ki-67 proliferation index was approximately 40%, and EBER *in situ* hybridization was negative.

**Figure 4:**
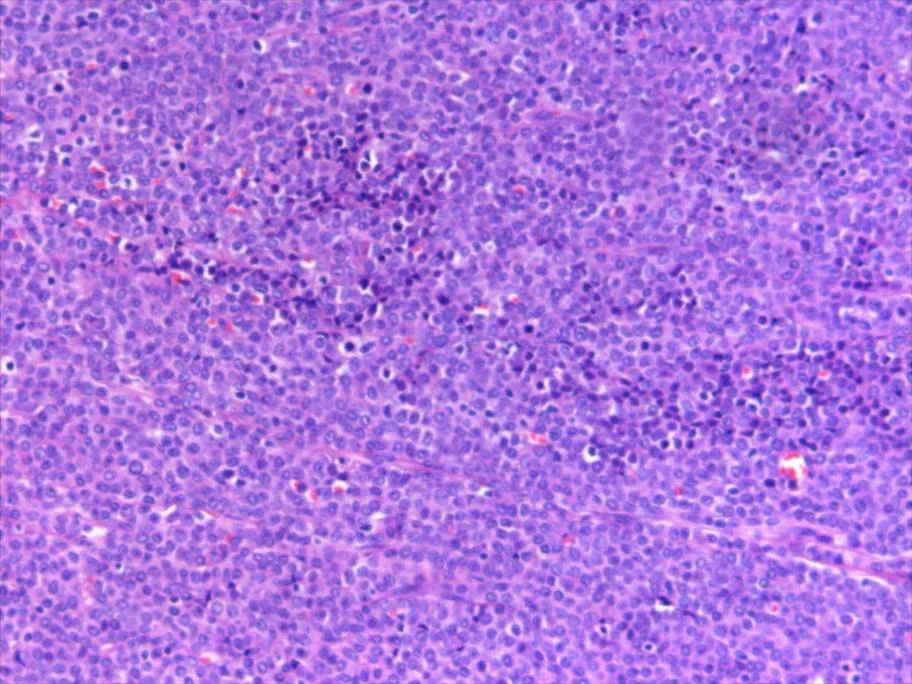
Histological report of an intraspinal mass consistent with a myeloid sarcoma or leukemic infiltration. Further blood and bone marrow examination were recommended.

Regarding the postoperative bone marrow aspiration (**Figure 5**), the findings indicated a hypercellular marrow with a myeloid-to-erythroid ratio of 2.47:1. The analysis revealed a relative increase in the myeloid lineage (60.5%) characterized by a predominance of mature forms, alongside a markedly increased number of megakaryocytes (>100 per smear) with large platelet clusters.

Regrettably, cytogenetic and molecular analyses (including FLT3, NPM1, RUNX1, and KIT) could not be conducted due to institutional resource limitations and the patient’s economic situation. The patient also refused postoperative MRI reevaluation due to financial constraints and a prolonged hospital stay.

On the second postoperative day, the patient reported marked relief of left lower extremity pain and paresthesia. In this case, the patient recovered uneventfully after tumor resection and was immediately transferred to the hematology department of an external hospital for comprehensive treatment, including systemic chemotherapy. A six-month postoperative follow-up was conducted, during which no recurrence of spinal cord compression symptoms was observed. However, MRI surveillance after discharge revealed disease progression, indicating a poor prognosis. The external hospital MRI reporter showed the following findings: Abnormal signal intensities in the right cerebellar hemisphere and the left frontal lobe, suggestive of metastatic lesions; the left frontal lobe lesion was slightly enlarged compared with the October 2023 images, while the right cerebellar lesion remained essentially unchanged. Status post internal fixation at L1–L3 vertebrae, with local intraspinal abnormal signals—differential considerations include metastasis versus artifacts; clinical correlation is advised. An intraspinal space-occupying lesion extending from the inferior margin of L4 to the S2 vertebral level, with thickening and local enhancement of the left nerve root, and osseous destruction at S1, all consistent with metastatic disease and showing slight progression compared with prior studies. Heterogeneous decreased signal intensity on T1-weighted imaging involving the clivus, visualized cervical and lumbar vertebrae, several vertebral bodies, and the iliac bones—clinical correlation and follow-up are recommended. Soft tissue swelling in the lumbar subcutaneous region.

Unfortunately, subsequent follow-up contact with the family ascertained that the patient had passed away in April 2024. A schematic illustrating the patient’s clinical course has been prepared (**Figure 5**).

**Figure 5:**
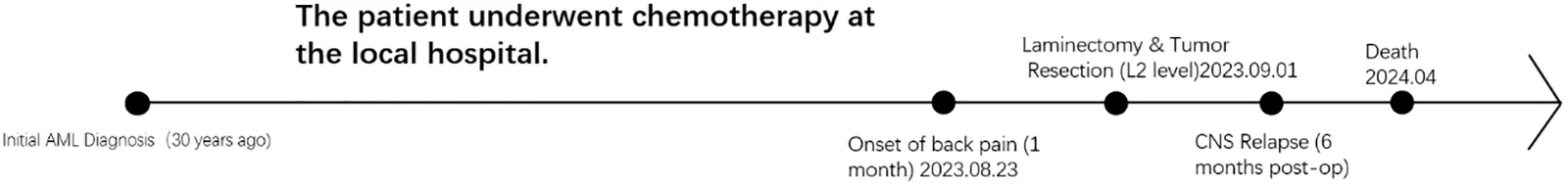
Schematic representation of the patient’s clinical course.

## 3 Discussion

Myeloid sarcoma is a distinct extramedullary tumor composed of myeloid blasts or immature myeloid cells. Although it was first described by Burns in 1811, the term ‘chloroma’ (from the Greek ‘chloros’ for green) was coined by King in 1853 based on his detailed postmortem investigations; he observed the tumor’s distinctive green color, noted that it faded upon exposure to air, and associated these lesions with underlying leukemia (2). In 1966, Rappaport proposed the term ‘granulocytic sarcoma.’ The current nomenclature, ‘myeloid sarcoma,’ was formally adopted by the World Health Organization (WHO) in 2002 (3). In the most recent updates released in 2022 by both the WHO (4) and the International Consensus Classification (ICC) (5), myeloid sarcoma retains its definition as a distinct entity within the spectrum of myeloid neoplasms.

It is worth noting that the alternative name ‘chloroma’ (green tumor) originates from the high expression of myeloperoxidase (MPO) in myeloid sarcoma. This high MPO content causes the cut surface of the tumor to appear green and fade upon exposure to air, hence the name (6).

Clinically, GS can be stratified into four specific contexts (7): 1) isolated or *de novo* GS, occurring in the absence of bone marrow involvement; 2) concurrent GS, presenting simultaneously with a diagnosis of AML; 3) blast phase GS, acting as a harbinger of leukemic transformation in patients with antecedent myeloproliferative neoplasms, myelodysplastic syndromes, or chronic myeloid leukemia (8); and 4) relapsed GS, appearing as a recurrent extramedullary disease following chemotherapy or hematopoietic stem cell transplantation.

Previous studies (9, 10) indicate that GS accounts for 2.5%–9.1% of patients with AML. No significant age-specific predisposition has been established, with its distribution relatively balanced across age groups. The incidence of GS is significantly higher in male patients than in females, showing a distinct male predilection. This sex difference may be related to the interaction of multiple factors such as genetics, hormone levels, and immune microenvironment; however, its exact mechanism requires further in-depth research for precise explanation and analysis (11). This will deepen the understanding of the pathogenesis behind this disease’s sex disparity and provide a crucial theoretical basis and practical guidance for optimizing subsequent personalized diagnosis and treatment strategies.

The diagnosis of GS is a persistent dilemma in clinical practice, with reported misdiagnosis rates of 25%–47%. Due to its morphological heterogeneity, GS is prone to being mistaken for other malignancies, including Hodgkin’s and non-Hodgkin’s lymphomas, especially in cases with poorly differentiated blasts. In our review of the literature regarding spinal myeloid sarcoma from the past decade (12–27), we found that spinal cord compression is the predominant initial presentation, mirroring the current case. Clinical symptoms typically include dorsal and lower extremity pain, motor weakness, and autonomic dysfunction (**Table 1**).

**Table 1:** Summary of reported cases of spinal myeloid sarcoma.

Case number	Sex	Age (years)	Clinical presentation	Imaging findings	Therapeutic approach	Patient outcomes
Case 1 (12)	Male	30	Low back pain	Magnetic Resonance Imaging (MRI) revealed an enhancing soft tissue mass in the paravertebral region extending from T5 to T8 (right side greater than the left), with involvement of the T5, T6, and T7 vertebral bodies. Spinal canal stenosis was observed at the T6 level due to the presence of the soft tissue mass.	Decompressive surgery with mass resection. Bone marrow transplantation with chemotherapy.	Survived after 1-year of follow-up
Case 2 (13)	Male	43	Pain in the neck, right shoulder, and arm for approximately 2 weeks.	Cervical spine X-ray shows mild multilevel degenerative changes with osteophytes noted at C5, C6, and C7 levels, although the disc height is relatively well-preserved. Cervical MRI reveals an enhancing epidural soft tissue mass extending from C7 to T1, with severe compression of the thecal sac at the T1 level.	Posterior cervical open biopsy, laminectomy, and tumor decompression	Not reported
Case 3 (14)	Male	24	Recurrent pain in the right leg and hip persisting for 6 months, with gait disturbance for nearly 1 month.	Spinal MRI reveals an intraspinal mass lesion extending from L5 to S2. The lesion demonstrates intermediate signal intensity on both T1-weighted and T2-weighted images and shows relatively homogeneous enhancement following contrast administration. The mass extends from the sacral foramina into the right paravertebral soft tissues, with associated destruction of the adjacent bone also noted.	L5–S2 decompression with hemilaminectomy and chemotherapy	Not reported
Case 4 (15)	Male	57	A tingling sensation radiating from the right arm to the axilla, along with a 7-month history of progressive weakness of the right upper limb and numbness in the right hand.	Magnetic Resonance Neurography (MRN) demonstrates marked thickening extending from the cervical nerve roots to the right brachial plexus, suggestive of a neoplastic process. FDG-PET/CT reveals hypermetabolic lesions in the right brachial plexus and the sacrum. Lumbar spine Magnetic Resonance Imaging (MRI) shows a mass lesion in the sacrum with extension into the adjacent soft tissues.	Chemotherapy and Allogeneic Bone Marrow Transplantation	The patient remains in remission 20 months after diagnosis.
Case 5 (16)	Male	55	The patient developed progressive weakness in both lower extremities 10 months after receiving two cycles of induction chemotherapy and subsequent allogeneic stem cell transplantation.	Lumbar spine magnetic resonance imaging (MRI) demonstrates a large T2-hyperintense lesion. This lesion extends into the dorsal epidural space and the paravertebral soft tissues, causing severe compression and flattening of the thecal sac at the L2-L3 level with associated traction on the cauda equina nerve roots. T1-weighted sagittal sequences reveal multiple T1-hypointense lesions within the vertebral bodies. Post-contrast T1-weighted sagittal and axial images show extension of the lesion into the L2-L3 neural foramina with enhancement, findings consistent with myeloid sarcoma.	Following the failure of stem cell transplantation and with the disease having progressed to an advanced stage, the patient has opted for palliative care	Not reported
Case 6 (17)	Male	24	Rapidly progressive tetraplegia for over 1 week, presenting with inability to walk and difficulty urinating.	Sagittal T2-weighted and contrast-enhanced T1-weighted magnetic resonance imaging (MRI) of the cervical, thoracic, and lumbar spine reveal multiple extra-axial masses involving the cervicothoracic region (from C5 to T3) and the lumbosacral region (from L5 to the coccyx). These lesions demonstrate moderate, homogeneous enhancement after contrast administration. Axial post-contrast T1-weighted images show that the tumor burden is most severe at the C6-C7 level in the cervical spine, with bilateral neural foraminal involvement and significant compression of the spinal cord. The intrathecal space below L5 is completely occupied by tumor. Furthermore, there appears to be bone remodeling involving the sacrum and the cervical neural foramina. Brain MRI shows no evidence of residual mass or enhancement in the right cerebellopontine angle, and a postsurgical change from a retrosigmoid craniotomy is noted on the left side.	Following administration of high-dose corticosteroids, the patient exhibited improvement in neurological function, with motor examination improving to 4+/5 strength and sensory deficits showing resolution. The patient was able to ambulate with minimal assistance	Seventeen months later, the patient was readmitted due to recurrence of quadriplegia. Thoracic spine MRI demonstrated tumor recurrence at the same location as the previously identified thoracic lesion. Twenty-nine months later, the patient presented again to the emergency department with headache, dysarthria, and lethargy. Brain MRI revealed a large mass in the right cerebellopontine angle (at the same location as the initial tumor) along with smaller masses in the cerebellar and pineal regions. Following radiotherapy, the patient was referred to the Medical Oncology department for follow-up and chemotherapy.
Case 7 (18)	Male	14	Headache and urinary incontinence for several weeks.	Brain and spine magnetic resonance imaging (MRI) reveals diffuse bone marrow infiltration involving the skull, facial bones, clivus, and cervical vertebrae. There is also a 2.1 cm epidural mass causing compression of the posterior third of the superior sagittal sinus. Brain MRI additionally demonstrates an extracranial mass. Spine MRI shows an infiltrative soft tissue mass in the epidural space extending caudally from the L5-S1 level. This mass fills the distal spinal canal, extends into the bilateral L5-S1 and all visualized sacral neural foramina, and is associated with mild thickening and enhancement of the distal cauda equina nerve roots. The imaging characteristics of the mass are suggestive of a chloroma (isointense on T1-weighted images, hyperintense on T2-weighted images, and demonstrates contrast enhancement). Chest X-ray findings are within normal limits.	Management included multimodal therapy with chemotherapy, radiotherapy, and stem cell transplantation	The patient remains in remission 7 months after hematopoietic stem cell transplantation, however, with chronic symptoms of a neurogenic bladder characterized by urinary retention and overflow incontinence.
Case 8 (19)	Male	68	The patient presented with low back pain and radiating pain in both legs, along with reported numbness in both feet, which rapidly progressed to paraplegia.	Thoracolumbar CT demonstrates mild discogenic endplate degeneration at T7-8, a small Schmorl’s node on the superior endplate of T12, and diffuse disc bulging from L1 to S1 with ligamentum flavum hypertrophy. Thoracolumbar MRI reveals a T2-hyperintense lesion in the epidural and paravertebral spaces at T11-T12, showing restricted water diffusion, which causes spinal cord compression at the T10-T12 level. These imaging features are suggestive of a chloroma (granulocytic sarcoma). Additionally, hemangiomas are detected at T12 and L4.	Emergency total laminectomy and excision of an epidural spinal tumor were performed for decompression. This was followed by palliative radiotherapy targeting the T9–T12 levels	The patient was discharged 35 days postoperatively and transferred to a local hospital for continued conservative management.
Case 9 (20)	Female	40	The patient reports numbness in both legs, and a firm, 3-cm mass in the upper outer quadrant of the right breast, present for two weeks. Hypoesthesia is noted below the T9 level.	Magnetic resonance imaging (MRI) demonstrates an intraspinal extra-axial solid lesion at the T9-T10 level.	The patient underwent a series of procedures and treatments including: right oophorectomy; excision of a mass in the lower outer quadrant of the right breast; total laminectomy at the T9-T10 level with subsequent lumpectomy; DA and EA therapy; prophylactic intrathecal chemotherapy; MA therapy; and radiotherapy	May 2004: A right oophorectomy was performed. Pathological diagnosis confirmed lymphoma. July 2004: She underwent excision of a mass in the lower outer quadrant of the right breast at another hospital. August 2004: Re-examination at our hospital led to a diagnosis of granulocytic sarcoma (GS). October 2017: The patient reported a chest mass persisting for two months. January 2018: Ultrasound examination revealed no chest mass. October 2019: The patient experienced intermittent chest pain for one month. A whole-body ^18^FDG-PET-CT scan identified a mass anterior to the anatomical body of the sternum. March 2020: Follow-up ultrasound showed no mass anterior to the sternum.
Case 10 (21)	Male	47	The patient reports a 6-month history of back pain radiating to the right lower extremity, accompanied by poor appetite and an approximate 5-kilogram weight loss.	Computed tomography (CT) of the chest, abdomen, and pelvis demonstrates an ill-defined osteolytic lesion with a soft tissue component, measuring approximately 112 × 86 × 155 mm, involving the left L4 to S5 vertebrae and invading the adjacent paravertebral muscles.	Chemotherapy	At the 24-month follow-up, all three patients reported complete resolution of back pain and lower extremity weakness. X-rays demonstrate maintained overall spinal alignment with no evidence of disease recurrence. Magnetic resonance imaging (MRI) shows no signs of compressive or residual lesions.
Case 11 (21)	Male	24	The patient has developed paralysis of both lower extremities over the past 15 days, accompanied by sensory loss in both lower limbs.	Spinal magnetic resonance imaging (MRI) reveals a lesion involving altered bone marrow signal intensity in the D4 through D7 vertebral bodies.	A D4-D7 laminectomy was performed, followed by radiotherapy	
Case 12 (21)	Female	32	The patient has experienced bilateral lower extremity weakness over the past two months, presenting with lower extremity paresis.	Spinal magnetic resonance imaging (MRI) reveals altered bone marrow signal intensity in the D6 through D8 vertebral bodies.	The patient underwent treatment involving chemotherapy, followed by a D6-D8 laminectomy, and subsequent radiotherapy	
Case 13 (22)	Male	25	The initial presentation was mid-back pain for two months, which was followed by the development of mid-back pain, chest pain, shortness of breath, and progressive paraparesis.	Chest X-ray reveals pleural effusion and collapse of the T8 vertebral body. Thoracic spine MRI demonstrates a posterior epidural space-occupying lesion at T7-T9 causing spinal cord compression. Both lumbar spine X-ray and lumbar spine MRI show an additional, relatively small and asymptomatic lesion at the L3 vertebral body.	A T8-T9 laminectomy for decompression and exploration was performed. The procedure included culture/biopsy of the lesion, epidural debridement, followed by T6-T10 pedicle screw fixation; chemotherapy was subsequently administered	One year after completing chemotherapy, the patient remains asymptomatic with no recurrence of the EMS
Case 14 (23)	Male	47	The patient reports left lower back pain radiating to the left lower extremity, accompanied by left lower extremity weakness and hypoesthesia for approximately nine months.	Lumbar MRI demonstrates a space-occupying lesion within the spinal canal at the L4-L5 level, which exhibits homogeneous enhancement along the L5 nerve root, consistent with a schwannoma. Brain MRI, as well as CT scans of the chest, abdomen, and pelvis, revealed no evidence of any space-occupying lesions or metastases.	An L4/L5 laminectomy was performed with tumor debulking	Follow-up MRI at 6 months postoperatively demonstrates a significant reduction in tumor size. The patient reports marked improvement in symptoms.
Case 15 (24)	Male	29	The patient experiences severe radicular pain in the lower extremities, accompanied by moderate dysuria.	Lumbar MRI demonstrates an isointense lesion within the spinal canal at the L2-L4 level, resulting in spinal cord compression. Spinal CT reveals an isodense, fusiform soft tissue lesion at L2-L4 with mild associated bone destruction. On fused SPECT/CT images, there is abnormal ^99m^Tc-MDP (methylene diphosphonate) accumulation in the L3 lamina and spinous process, with no evidence of other bone destruction or metastatic lesions identified.	L2-L4 pedicle screw instrumentation and fusion was performed; chemotherapy was administered	At the 10-month follow-up, the patient remains in good health with no evidence of acute myeloid leukemia (AML) involving the bone marrow and no recurrence of the local lesion.
Case 16 (25)	Female	50	The patient presents with paresthesia in the right hand and difficulty walking for one week.	Cervical MRI demonstrates a space-occupying lesion within the spinal canal at the C6-C7 level. The mass appears hypointense on T1-weighted images, iso-intense on T2-weighted images, and shows heterogeneous enhancement after contrast administration.	Resection of the intraspinal tumor with spinal fixation was performed; chemotherapy was administered	At the 10-month follow-up, the patient remains in complete hematologic and molecular remission.
Case 17 (26)	Male	20	The patient presents with numbness and weakness in both lower limbs, resulting in the inability to walk or even stand, and has had no urination for 12 hours.	Thoracic MRI reveals compression of the upper thoracic spinal cord from T2 to T6 due to a posteriorly located extradural/intradural neoplastic mass. The compressed spinal cord appears narrowed in width, with no evidence of vascular thrombosis or intramedullary/extramedullary hemorrhage.	Spinal decompression surgery was performed. A soft tissue mass compressing the spinal cord from T2 to T6 within the epidural space was excised; chemotherapy was administered	Not reported
Case 18 (27)	Male	61	The patient reports low back pain and difficulty walking for six months.	Spine MRI demonstrates a pathological fracture of the third lumbar vertebra (L3), along with multiple areas of abnormal signal intensity involving the left iliac bone and the vertebral bodies of the thoracic (T11-T12), lumbar (L1-L4), and sacral (S1-S3) spine.	Chemotherapy	MRI shows significant reduction in the spinal MS lesion, with resolution of the patient’s low back pain and walking difficulty. More importantly, the PLZF::RARα fusion transcript is no longer detectable, indicating achievement of complete molecular remission (MR). The initiation of any additional treatment will depend on successful recovery of the patient’s hematopoietic function.

The imaging differential diagnosis for intradural extramedullary GS includes both neoplastic and inflammatory conditions. Spinal infections typically involve vertebrae and discs with endplate erosion and paravertebral abscess, unlike isolated intradural GS. Inflammatory demyelinating diseases usually present as intramedullary rather than intradural extramedullary lesions. Sarcoidosis may cause leptomeningeal nodules with systemic findings. Granulomatous lesions often show rim enhancement and central necrosis, while GS is usually well circumscribed (27). When imaging is inconclusive, histopathology with immunohistochemistry is essential for diagnosis. We also performed a differential diagnosis of other intradural extramedullary intradural extramedullary masses and compared their typical imaging manifestations (28–35) (**Table 2**).

**Table 2:** Imaging differential diagnosis of intradural extramedullary spinal lesions.

Differential diagnosis	Common sites	CT findings	MRI findings (T1WI/T2WI)	CT Enhanced scan	Key distinguishing features
Schwannoma (28)	Slightly more in the lumbar segment, followed by the cervicothoracic segment.	The tumor density is slightly higher than that of the spinal cord, appearing round or oval-shaped. The corresponding spinal cord is compressed and displaced. The tumor tends to grow toward the intervertebral foramen. When the tumor extends through the dural sac along the nerve root sheath into the epidural space, it can form a dumbbell-shaped mass with both intrathecal and extrathecal portions.	T1WI: Usually manifests as Equal signal or slightly lower signal, Signal homogeneity is related to the size and internal structure of the tumor. Small schwannomas tend to have more homogeneous signals, whereas larger tumors may exhibit heterogeneous signals. Cystic changes and necrotic areas within the tumor appear as low-signal regions on T1-weighted imaging (T1WI); hemorrhagic areas may show high signal, particularly during the subacute phase; calcified areas, on the other hand, appear as low signal. T2WI: Typical manifestation is obvious. High signal, A small number of schwannomas may manifest solely as moderately high signal intensity. Approximately 60-70% of schwannomas exhibit a characteristic “target sign” on T2-weighted imaging, in which the central portion of the tumor appears as a high signal (Antoni B zone), while the surrounding area shows a relatively low signal (Antoni A zone). Clearly visible on T2WI Show the relationship between the schwannoma and its originating nerve. At the junction between the nerve and the tumor, the appearance may be characterized by: “Separation Syndrome,” that is, GodThe fibers are separated by the tumor; or “Entry and Exit of the Divine,” or simply, the Divine. Fibrous tissue from the swelling Both ends of the tumor Enter and exit.	Obvious And continuous Strengthen, The enhanced pattern exhibits “fast in, slow out”; heterogeneous enhancement is visible within the large tumor; 30-40% Schwannoma In CT enhanced scanning Possible Show characteristic features Target sign It manifests as prominent peripheral enhancement with relatively weaker enhancement in the central portion.	Along the divine Neurofibromas along the course of the nerve exhibit characteristic features. “Dumbbell sign” or the “boot sign,” especially in spinal nerves Schwannoma, which can traverse the intervertebral foramen along the nerve root, forming a dumbbell-shaped tumor both within and outside the spinal canal.
Neurofibroma (29, 30)	Slightly more in the lumbar segment, followed by the cervicothoracic segment.	The mass exhibits isodense or slightly hypodense characteristics with well-defined borders; in some cases, surrounding tissues may show compression and displacement. If cystic changes or necrosis occur within the tumor, areas of low density may appear.	T1WI: Low signal T2WI: Visible “target sign” The lesion appears as a low signal at its center, surrounded by a ring of high signal.	Moderate to marked enhancement, which may exhibit a “target sign”-like enhancement pattern.	It has MRI findings similar to those of schwannomas, but it more commonly grows along the perineural sheath.
Spinal Meningioma (31, 32)	Thoracic spinal cord	The lesion density is slightly higher than that of the spinal cord. The tumors are mostly solid, oval or round in shape, and most are confined to the posterior aspect of the thoracic subarachnoid space. They have a well-defined capsule, and occasionally irregular calcifications can be seen within the tumor mass.	T1WI: Isosignal When accompanied by calcification, it appears as low signal. After injection of Gd-DTPA, the tumor shows up as high signal on T1-weighted imaging (T1WI). Persistent homogeneous enhancement, with “tail-like” linear enhancement visible adjacent to the dura mater. T2WI: Isointense or slightly hyperintense signal When accompanied by calcification, it appears as low signal.	After contrast enhancement, the lesion shows moderate enhancement. CT myelography can reveal partial or complete obstruction of the subarachnoid space at the tumor site, with compression and narrowing of the spinal cord, as well as displacement toward the healthy side.	More common in women; Neural foramina rarely show enlargement, and dumbbell-shaped tumors are significantly less common than schwannomas.
Paraganglioma (33)	Extradural Subarachnoid Cauda Equina Region and Filum Terminale部位	The lesion appears isodense.	T1WI: Isosignal T2WI: Wait or High signal	Obvious Uniform strengthening	Enhanced vascular shadows with tortuous courses are visible on the upper and lower planes of the tumor.
Metastasis (34, 35)	Commonly occurs in the lumbosacral region.	The plain scan can be negative.	In the diffuse type, tumors can be seen enveloping the surface of the spinal cord or nerve root sheaths, causing linear enhancement; in severe cases, the entire dural sac may show shell-like enhancement. In the localized type, multiple nodular enhancements can be observed on the arachnoid membrane.	Enhanced scanning is more sensitive in detecting leptomeningeal metastasis.	A history of malignant tumors, often accompanied by other symptoms.

### 3.1 Common investigative modalities for GS

#### 3.1.1 Blood and bone marrow routine examination

Primary GS generally shows no abnormalities in bone marrow or routine blood tests. If tumor invasion causes bleeding or jaundice in organs, related changes may be seen in routine blood tests.

#### 3.1.2 Imaging examination

With the widespread use of Computed Tomography (CT) and MRI, these methods are also widely used in investigating cases of GS. However, imaging findings are non-specific, and no diagnostic imaging criteria have been established. Imaging findings are primarily used to determine the location and size of the tumor.

#### 3.1.3 Histopathological examination

Pathological examination of the mass remains the only definitive diagnostic method. Common sites for extramedullary infiltration include the skull and orbit, as well as the breasts, liver, kidneys, and muscles, among others. Myeloid sarcoma can also occur inside or outside lymph nodes. In GS, due to the high concentration of myeloperoxidase (MPO) within granulocytes, the cut surface of the tumor tends to oxidize in air and appear green; it is also called chloroma or green tumor.

### 3.2 Treatment methods

If isolated myeloid sarcoma is left untreated, 88% of patients will progress to AML within 1 year. The treatment of GS depends primarily on the location of the myeloid sarcoma, whether it is newly diagnosed or recurrent, its clinical presentation, and the patient’s age. Systemic chemotherapy plays a crucial role in the treatment of myeloid sarcoma. Even if there is no bone marrow involvement at onset, myeloid sarcoma should be treated as a systemic disease with systemic chemotherapy.

GS involving the spinal cord typically occurs outside the spinal cord (extradural or intradural extramedullary), presenting as spinal cord compression due to the mass effect; however, a small number of intramedullary cases have also been reported.

GS involving the spinal cord is rare. Without a history of related leukemia, its diagnosis is quite difficult. MRI is an important method for diagnosing this condition, requiring differentiation from lymphoma, metastatic tumors, extramedullary hematopoiesis, and neurogenic tumors.

MRI findings of multiple or consecutive extramedullary masses combined with diffuse abnormal bone marrow signals can help in the preliminary diagnosis of leukemia complicated with spinal GS. GS appears isointense or slightly hyperintense on T2-weighted MRI images and isointense on T1-weighted images.

Due to the rarity of spinal cord-involving GS, there is no unified treatment model. Treatment mostly involves surgical decompression, chemotherapy, radiotherapy, stem cell transplantation, or combinations of these modalities. Most scholars believe that GS requires comprehensive treatment combining chemotherapy with surgical resection or radiotherapy. For isolated spinal GS, the optimal sequence of surgical resection and radiotherapy remains debated. Surgical decompression is prioritized for acute neurological deficits, as in our case, to preserve function. However, early initiation of systemic chemotherapy is paramount to eradicate micrometastatic disease and prevent systemic relapse or progression to AML. Surgical intervention is primarily indicated for patients with acute spinal cord compression and neurological symptoms. Microsurgical resection of the tumor generally leads to significant improvement in neurological symptoms and enhances the patient’s quality of life.

The prognosis of spinal GS is generally guarded and hinges on several factors. Favorable predictors include isolated *de novo* presentation, achievement of complete surgical resection when feasible, and prompt, effective systemic therapy leading to sustained remission. As illustrated by the present case, even with successful initial surgical and chemotherapeutic intervention, the risk of disseminated CNS relapse remains significant, highlighting the aggressive nature of this disease.

### 3.3 Patient perspective

The patient reported that the pain and numbness in his left leg improved after surgery. This symptomatic relief contributed to a perceived enhancement in his postoperative quality of life.

### 3.4 Limitation

In this case, we diagnosed the patient with spinal granulocytic sarcoma based on preoperative imaging, the patient’s medical history, intraoperative findings, and postoperative pathological examination. However, due to the limitations of our hospital’s resources and the patient’s economic situation., we were unable to perform cytogenetic examination, which precluded a precise diagnosis. This constitutes a shortcoming of the present case report.

## 4 Conclusion

This case offers critical insights into the management of spinal myeloid sarcoma, underscoring the inherent diagnostic complexity and therapeutic challenges of this rare entity. By detailing the trajectory from initial presentation and radiographic ambiguity to definitive histopathological confirmation, this report highlights the importance of a multidisciplinary approach. Such collaboration is essential to minimize diagnostic latency, prevent misidentification, and ensure the timely initiation of precise, systemic therapy.

The surgical intervention performed at our hospital effectively alleviated the symptoms of spinal cord compression, underscoring the critical role of surgery in acute neurological management. However, despite the initial benefit, the patient’s eventual poor prognosis, notwithstanding the unavailable details of his subsequent care at an outside institution, highlights the aggressive nature of spinal myeloid sarcoma and the significant limitations of current therapeutic modalities.

Given the rarity of myeloid sarcoma and the absence of a standardized therapeutic consensus, large-scale, multicenter collaborative studies are imperative. Such efforts are essential to evaluate the efficacy and safety of diverse treatment modalities, elucidate underlying pathological mechanisms, and establish precise, personalized therapeutic algorithms. Ultimately, bridging current therapeutic gaps through rigorous research is paramount to optimizing clinical management and improving survival outcomes for this challenging patient population.

## Data Availability

The original contributions presented in the study are included in the article/**Supplementary Material**. Further inquiries can be directed to the corresponding author.
